# Extracellular vesicles as circulating cancer biomarkers: opportunities and challenges

**DOI:** 10.1186/s40169-018-0192-7

**Published:** 2018-05-31

**Authors:** R. E. Lane, D. Korbie, M. M. Hill, M. Trau

**Affiliations:** 1 Australian Institute for Bioengineering and Nanotechnology, The University of Queensland, St Lucia, QLD Australia; 2 The University of Queensland Diamantina Institute, Faculty of Medicine, Translational Research Institute, The University of Queensland, Woolloongabba, QLD Australia; 3 QIMR-Berghofer Medical Research Institute, Herston, QLD Australia; 4 School of Chemistry and Molecular Biosciences, The University of Queensland, St Lucia, QLD Australia

**Keywords:** Exosome, Microvesicle, Extracellular vesicle, Cancer, Biomarker

## Abstract

Extracellular vesicles (EVs) are small, lipid-bound particles containing nucleic acid and protein cargo which are excreted from cells under a variety of normal and pathological conditions. EVs have garnered substantial research interest in recent years, due to their potential utility as circulating biomarkers for a variety of diseases, including numerous types of cancer. The following review will discuss the current understanding of the form and function of EVs, their specific role in cancer pathogenesis and their potential for non-invasive disease diagnosis and/or monitoring. This review will also highlight several key issues for this field, including the importance of implementing robust and reproducible sample handling protocols, and the challenge of extracting an EV-specific biomarker signal from a complex biological background.

## Introduction

Extracellular vesicles (EVs) have garnered much recent interest due to their potential utility as circulating biomarkers for cancer. EVs have been implicated in a diverse range of physiological functions due to their capacity to convey protein and nucleic acid species from a donor cell to a recipient. Tumour-derived EVs have been demonstrated to carry disease-associated molecular cargo, and further, observed to modulate the behaviour of recipient cells towards a pro-oncogenic phenotype. The correlation between the tumour cell and tumour-EV proteome and transcriptome across multiple tumour contexts has highlighted the potential for tumour-EVs as candidate markers for disease diagnosis and monitoring. This review summaries the current understanding of EV form and function in the context of cancer, highlighting key and transformative works in this space. We also discuss some of the current limitations in this field, and the challenges to address for EV biomarkers to have clinical utility.

## Overview of extracellular vesicles (EVs)

Extracellular vesicle is a general term used to describe cell-derived sub-micron membranous vesicles which are released into the extracellular space. Following release, EVs can enter the circulation and have been isolated from numerous bodily fluids including blood [1], urine [2], saliva [3], ascites [1] and breast milk [4]. This review will consider two major EV subclasses: exosomes, which are endosomally derived, and microvesicles (MVs, also referred to as ectosomes) which bud from the plasma membrane surface. Various other terms have been used to describe specific subsets of EVs, however, the general terms ‘exosome’ and ‘microvesicle’ are the most widely recognised within the field.

**Fig. 1:**
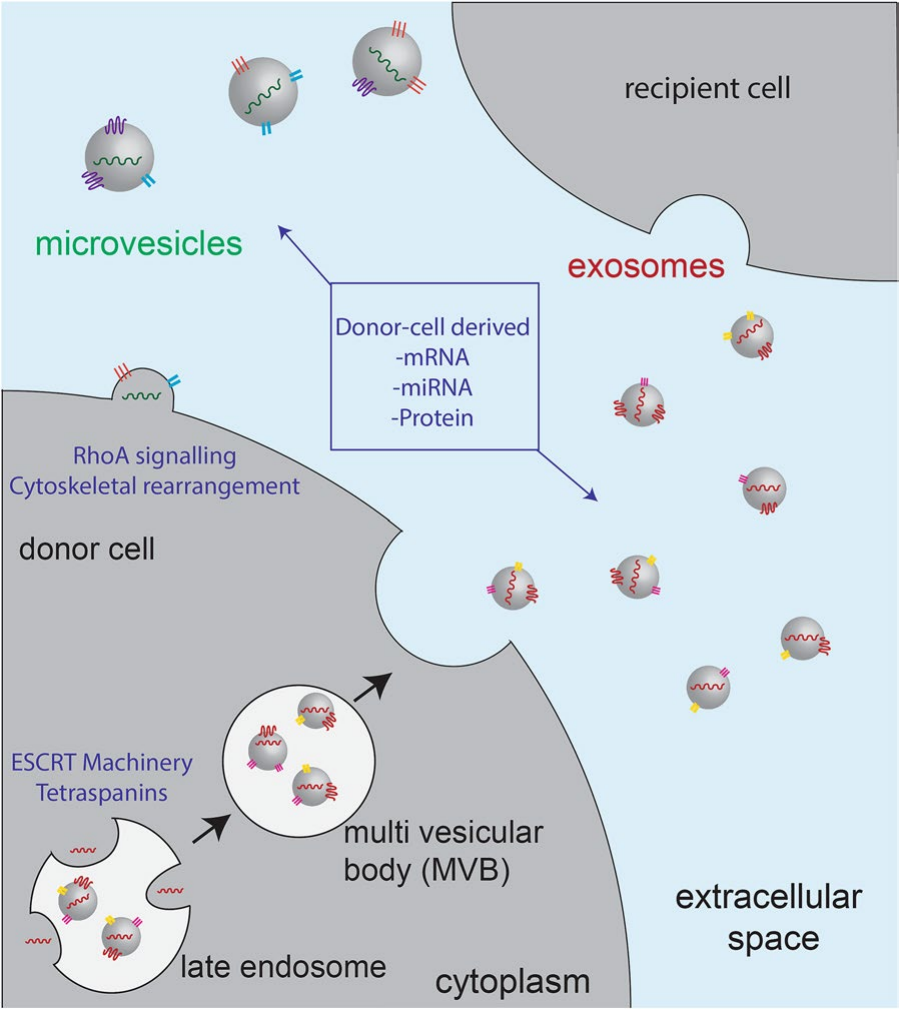
Schematic of the process of exosome and microvesicle secretion. Exosomes are endosomally derived, and bud inside an intermediate structure known as a multi-vesicular element (MVE). The MVE subsequently fuses with the plasma membrane of the cell, releasing the contents. Microvesicles bud directly from the plasma membrane surface, preceded by a rearrangement of the membrane lipid bilayer and the local cytoskeleton

It is generally understood that most cells release a mixture of both exosomes and MVs into the extracellular space [5, 6]. Given this, it can be difficult to reliably associate physical, molecular and functional properties with a specific vesicle subclass. This section will provide a brief summary of the current understanding of exosome and microvesicle formation and release, as illustrated in Fig. 1. The subsequent sections will consider both vesicle classes together, using the term ‘EVs’ to denote a mixed population.

### Exosomes

Endosomally-derived vesicles were first described in the early 1980’s during studies of reticulocyte maturation [7, 8]. This early work demonstrated that transferrin receptor shed from maturing reticulocytes in culture was associated with sub-200 nm vesicular structures [7]. Electron microscopy demonstrated that these vesicles arose within a larger endocytic cellular compartment termed a multi-vesicular element (MVE) [8, 9]. This MVE was then observed to fuse with the plasma membrane of the cell and release the small vesicles to the extracellular space [8, 9]. It was initially hypothesised that vesicle release in this manner was a reticulocyte-specific mechanism to shed unneeded protein material during maturation [10, 11]. Later studies during the 1990’s and early 2000’s, however, suggested that this phenomenon occurred across numerous haematic and non-haematic cell types, including B cells [12], dendritic cells [13], epithelial cells [14] and notably, tumour cells [15].

Exosomes are known to carry protein cargo specific to their cell of origin, however, they also appear to carry a core set of constituents including cytoskeletal proteins (e.g. actin, myosin), heat shock proteins (e.g. HSP70, HSP90), tetraspanins, and vesicular transport associated proteins (e.g. Rab, Annexin A2, Annexin A5) [16–18]. The mechanisms underlying exosomal cargo selection have yet to be fully elucidated, and appear to be modulated by a range of protein and lipid species. It appears that MVE formation and exosome budding is in part modulated by the endosomal complex required for transport (ESCRT) machinery, a system of five protein complexes involved in the reorganisation of cellular membranes [19–21]. Exosomal budding and cargo selection appears to be partially mediated by tetraspanins, a class of membrane spanning proteins [16, 22]. Certain members of the tetraspanin family, including CD9, CD63, CD81 and CD82 are used as conventional exosome markers, and are thought to be ubiquitously present on vesicles derived from various cellular sources [16, 22]. It is hypothesised that tetraspanin-enriched membrane microdomains within the MVE may facilitate the recruitment of specific protein cargo for inclusion in the resultant vesicles [17, 23]. There is also some evidence that exosome budding may be mediated by the presence and/or abundance of certain lipid species, although, this mechanism appears to be cell type specific. For example, Trajkovic and colleagues [24] reported that in oligodendrocyte precursor cells (Oli-neu), inhibition of ceramide formation decreased vesicle budding, however, this effect was not observed in either prostate cancer (PC-3) [25] or melanoma (MNT-1) cell lines [20] in separate investigations. In total, it is evident that there is specific selection of exosome cargo, and that this is regulated by multiple cellular mechanisms. A more detailed review of the process of exosome biogenesis and release has been presented by Hessvik and Llorente [26].

### Microvesicles

Microvesicles (MVs) were first described in the 1960’s by the observation that platelets released lipid-rich particles with pro-coagulant activity from the cell surface into their surroundings [27]. It was later discovered that this surface shedding, sometimes referred to as ‘ectocytosis’ [28], occurred across numerous cell types including monocytes [29], neutrophils [28], oligodendrocytes [30] and tumour cells. In the late 1990’s, Heijnen and colleagues [31] first observed that release of both microvesicles and endosomally-derived exosomes could arise from a single cell.

Microvesicles typically carry some of the plasma membrane components of the cell of origin, which can include integrins, selectins and/or tetraspanins [32]. The MV proteome, however, does not directly reflect that of the cell, implying that some selection of cargo occurs during vesiculation [32]. MVs are more physically heterogenous than exosomes, and are reported to range in size from 0.1 to 1 µm [33]. MVs also appear to carry a diverse range of protein cargo, and as such, a ubiquitous set of specific MV markers have yet to be clearly defined. The most commonly used marker is the lipid phosphatidylserine with proteins including integrin-β1, flotillin-1 and tissue factor proposed as candidates [33]. The sequence of events underlying MV release have been relatively well defined. MV release appears to be driven by an increase in intracellular Ca^2+^ levels which triggers a membrane rearrangement [34]. There is a simultaneous cytoskeletal rearrangement, initiated by transforming protein RhoA, and culminating in MV budding [35]. The process of budding and release of MVs and their potential role in tumorigenesis has been reviewed in detail by Surman and colleagues [33].

## Physiological roles of EVs

**Fig. 2:**
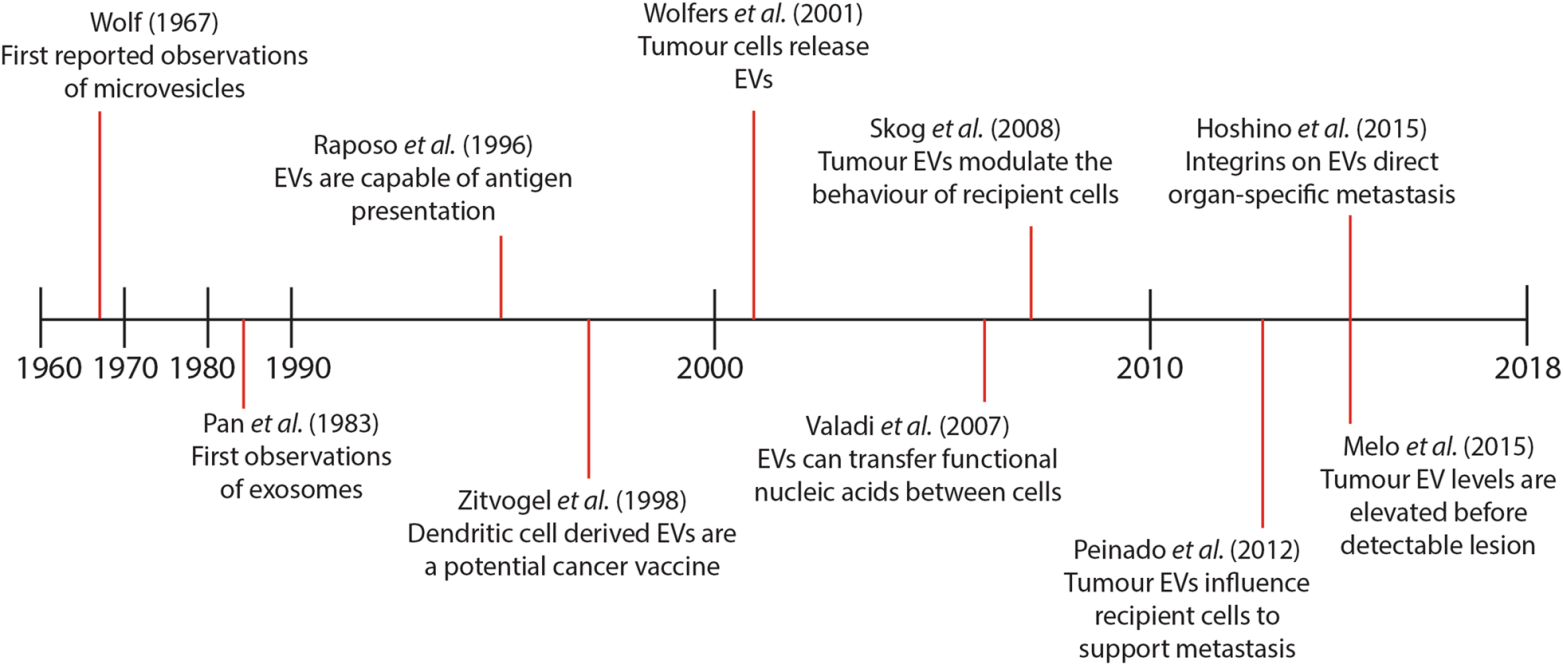
Timeline of key discoveries in extracellular vesicle research. Microvesicles were first reported in the 1960’s, and exosomes in the 1980’s. The physiological role of EVs in antigen presentation and cell–cell communication were first reported in the 1990’s and 2000’s respectively. From the late 2000’s onwards, several key works have highlighted the role of tumour EVs in promoting cancer growth and metastasis, and highlighted their potential utility as biomarkers

Despite initially being thought of as a mechanism for cellular waste removal, it has subsequently become apparent that EVs, including exosomes and microvesicles, play several important roles in normal and pathological physiology. EVs appear to have immunogenic properties, and can be involved in antigen presentation to immune effector cells [12]. Further, EVs have been implicated in cell–cell communication, and have been observed to transfer functional nucleic acids and proteins between cells [36]. This function appears to be particularly important in a disease context, and may represent a mechanism to promote tumour growth and metastasis [37]. The following section will discuss the current understanding of the key physiological functions of EVs. A timeline describing some of the most important discoveries in the field of EV research is included as Fig. 2.

### Immune-associated roles of EVs

In the mid-1990’s it was reported that EVs secreted from antigen presenting cells (APCs) appeared to have immunogenic properties. An early study by Raposo and colleagues [12] showed that EVs shed by B lymphocytes carried major histocompatibility complex class II (MHC-II) molecules on their surface, and were capable of inducing antigen-specific T helper cell responses. A subsequent investigation reported that EVs secreted from dendritic cells (DCs) contained both MHC-I and MHC-II molecules, and were similarly capable of inducing an immune response in vivo [13]. Wolfers and colleagues [15] later reported that tumour cells also secrete EVs bearing MHC-I molecules. These tumour-derived EVs were shown to transfer tumour antigens to DCs, enabling a T cell specific anti-tumour response in vivo [15]. In the context of infection, macrophages infected with *Mycobacterium bovis* were demonstrated to secrete EVs which could activate specific CD4^+^ and CD8^+^ T cell responses [38]. In total, there is a substantial body of evidence to suggest that EVs represent an important mechanism for communication between APCs and immune effector cells.

These findings stimulated interest in the potential to use DC-derived EVs as a component of an autologous cancer vaccine. An early study by Zitvogel and colleagues [13] using a mouse model demonstrated that DC-derived EVs were capable of inducing an anti-tumour response in vivo, slowing tumour growth and in some cases, completely eradicating an established tumour. Several phase I human trials were subsequently run, enrolling patients with melanoma [39] and non-small cell lung cancer [40] respectively. In both of these trials, EVs were produced from patient DCs, pulsed with antigenic peptides and injected. These treatments appeared to promote disease stabilisation in a few patients, however, the efficacy of DC-derived EVs has yet to be established for cancer immunotherapy [39–41]. The use of EVs as cancer immunotherapeutics is reviewed in depth in [41].

### EVs in cell–cell communication

In the mid-2000’s, it was postulated that EVs may represent a mechanism of cell–cell communication beyond their immunogenic capacity [42]. In a landmark study, Valadi and colleagues [36] demonstrated that EVs isolated from a mouse mast cell line could transfer functionally intact mRNA to be translated in human mast cells. The investigators also noted that the EVs appeared to carry several species of miRNA, and hypothesised these could also be transferred between cells [36]. These initial findings have since been recapitulated in numerous studies. For example, Gross et al. reported that EVs from *Drosophila melanogaster* and human cell lines carry Wnt proteins, a key class of morphogen, which are capable of activating downstream signalling pathways in recipient cells [43]. EV-mediated communication has also been implicated in several other key developmental processes, including early implantation [44], angiogenesis, and protection of the fetus and placenta from the maternal immune system [45].

### EVs in cancer

Despite the diverse roles of EVs in normal physiology, arguably the most well studied form EV-mediated communication has been in the context of tumour growth and metastasis. Skog and colleagues [37] were amongst the first to explore this phenomenon, reporting that EV from glioblastoma cells could upregulate angiogenic behaviour in normal brain endothelial cells, via the transfer of nucleic acid and protein. This phenomenon has since been observed across numerous tumour contexts, and several notable examples will be described here. In an in vitro model of hypoxia, Park et al. [46] observed that squamous carcinoma cells secrete proteins and EVs which together lead to decreased adhesiveness and increased angiogenic behaviour in recipient cells. In a later study, EVs secreted from a highly invasive variant of the HS578T (HS578Ts(i)_8_) breast cancer cell line were demonstrated to upregulate the proliferative, migratory and angiogenic potential of several recipient cell lines, including the parent cell line, in vitro [47]. This same phenomenon was later noted in an in vivo model of breast cancer [48]. Further, in contrast to the immunogenic capacity of DC-derived EVs, tumour-EVs appear to exert immunosuppressive effects [49]. Tumour-EVs have been observed to suppress the activity of natural killer cells [50, 51] and T cells [52] and to promote the differentiation of myeloid derived suppressor cells [53, 54]. This is postulated to contribute to immune tolerance of the tumour, and therefore inhibition of these tumour-EV activities has been proposed as a therapeutic strategy [53]. In total, these studies provide strong evidence that EVs are a mechanism of communication for tumour cells to promote proliferation, invasiveness and evasion of the host immune system.

In addition to influencing the local tumour environment, there is also evidence to suggest that EVs may be involved in initiating and/or supporting tumour metastasis at distant sites. It has been reported that EVs derived from the highly metastatic B16-F10 melanoma cell line could recruit bone marrow derived cells (BMDCs) to promote the establishment of metastatic lesions [55]. A similar mechanism was observed in a model of pancreatic ductal adenocarcinoma, whereby tumour-derived EVs were found to specifically interact with a subset of resident liver cells, inducing fibrosis and the recruitment of BMDCs to this site [56]. Another recent and noteworthy study examined the potential role of EVs in the phenomenon of metastatic organotrophism across a variety of cancer types [57]. This study implicated integrins on the EV surface as a key factor determining the establishment of pre-metastatic niche sites in specific organs [57]. Taken together, these reports indicate that tumour cells may employ EV-mediated communication to facilitate metastasis to distant sites.

## EVs as cancer biomarkers

There has been considerable interest in exploring the use of tumour-EVs for disease diagnosis and monitoring. It is generally understood that EVs contain nucleic acid and protein cargo representative of the secreting cell, and this has been established across a number of tumour contexts [58]. The presence of tumour-EVs in circulating bodily fluids such as blood, urine and cerebrospinal fluid means they represent a readily accessible source of biomarkers. This suggests they may have particular utility for longitudinal disease monitoring and early detection of relapse [59]. It has also been reported that certain EV-associated protein and nucleic acid species may be predictive of response to treatment. In total, there is a growing body of evidence that suggests EVs could represent a rich and accessible source of cancer biomarkers.

Amongst the first reports exploring the biomarker potential of tumour-EVs was a comparison of the content of glioblastoma EVs to their cells of origin [37]. Skog et al. [37] reported that EVs contained tumour-associated RNA and protein species that were a ‘snapshot’ of the content of the secreting cell. Subsequently, the presence of known cancer-associated miRNA [60], mRNA [37, 61], lncRNA [62] and post-translational protein modifications [63] in tumour-derived EVs has been established across numerous investigations. This phenomenon has been demonstrated in multiple cancer types, and was exemplified by a recent study by Hurwitz and colleagues profiling sixty cancer cell lines. The EV proteome was found to reflect the cellular proteome and transcriptome across all samples analysed. This was exemplified by hierarchical clustering based on the EV proteomic data, where samples were found to segregate based on the tissue type of the originating cell [58]. The correlation between tumour-EV and tumour cell content is particularly valuable where the ability to conduct a tissue biopsy is limited, such as in tumours of the brain and central nervous system. For example, studies of glioblastoma multiforme have indicated that tumour-EV in the cerebrospinal fluid contain elevated levels of miR21 relative to healthy controls, and that EV-miR21 levels reflect tumour burden [64, 65]. Prognostically informative tumour-EV miRNA signatures have similarly been identified in pancreatic cancer [66], colorectal cancer [67] and non-small cell lung cancer [68]. Similarly, in a study of Non-Hodgkin’s lymphoma patients, Provencio and colleagues [69] identified that the presence of several candidate mRNAs including C-MYC, BCL-6 and PTEN in plasma-derived EVs was predictive of progression free survival. These, and other reports, have suggested that tumour-EVs may therefore have potential for non-invasive longitudinal disease monitoring [70].

It has also been suggested that the nature of tumour-EV release may provide opportunities for early disease detection. Melo and colleagues [71] reported that in an in vivo model of pancreatic cancer, the level of EVs bearing a candidate marker protein was elevated prior to the tumour being detectable by conventional imaging techniques. Similarly, in a study of acute myloid leukaemia (AML), Hornick et al. [59] observed that AML-EVs were detected in the circulation prior to leukaemic blasts appearing in the blood. There is also evidence that tumour-EVs may have utility in predicting response to treatment. Tumour-derived EVs have been implicated in resistance to numerous therapeutics by mediating the transfer of specific miRNA and/or protein species from drug-resistant to drug-sensitive cells. This phenomenon has been demonstrated across several cancer types and therapies including Tamoxifen (anti-estrogen) [72] therapies in breast cancer, Cetuximab (anti-EGFR) therapy in colon cancer [73] and Pazopanib (chemotherapy) in soft tissue sarcoma [74]. In these studies, exposure to EVs from resistant cells was demonstrated to disrupt drug-associated signalling pathways in sensitive recipients, and this is proposed to contribute to the development of resistance. Notably, a distinct mechanism has also been described for Trastuzumab (anti-HER2) therapy in breast cancer [75]. EV-associated HER2 appears to be capable of binding this drug, thereby reducing the available concentration and diminishing the therapeutic effect [75]. In total, these observations have suggested that tumour-EV biomarkers have potential prognostic and predictive value.

## Current challenges

Whilst tumour-EVs represent a promising class of circulating biomarker, it is worthwhile to note some current limitations in this field of research. One major challenge for this field is the lack of standardisation of protocols for EV enrichment and characterisation. The use of disparate EV handling and analysis protocols means that reported sample characteristics can vary between studies, and this complicates inter-study comparisons. In response to this, the EV-TRACK knowledgebase (http://evtrack.org) [76] was recently established. This resource is designed to facilitate inter-study methodological comparisons and develop guidelines for experimental design and reporting in EV research.

The following section will outline some of the commonly used methods for EV enrichment and characterisation, highlighting specific issues associated with each. We also present a brief discussion of some of the major challenges for translation of tumour-EV biomarkers to the clinic.

### Isolation and enrichment of EVs

The use of appropriate sample handling methods is of particular importance for the biomarker potential of tumour-EVs to be realised. There is substantial evidence that the method of sample handling can impact the apparent physical and molecular characteristics of these samples [70]. Variability can be introduced by biases in the isolation or detection of certain EV components [77], or as contaminating artefacts which are not completely removed during sample processing [78]. Moreover, it has also been suggested that more thorough reporting of EV handling and measurement protocols is warranted, in order to facilitate inter-study comparisons and improve the reproducibility of results [76, 79, 80].

The main methods used to isolate EVs are differential ultracentrifugation, density gradient ultracentrifugation, polymer-facilitated precipitation (e.g. ExoQuick, Total Exosome Isolation Kit), immunoaffinity isolation and, size exclusion chromatography (SEC). The majority of EV investigations employ one or more of these methods as part of an isolation workflow. Developing an appropriate workflow is dependent on the starting material, required purity of isolates and available equipment. The relative advantages and limitations of various EV isolation protocols have been the subject of several previous reports, and will be briefly summarised here.

Differential ultracentrifugation is arguably the ‘gold standard’ for EV isolation. This method, initially described by Théry and colleagues [81], involves a series of sequential centrifugation steps designed to enrich < 200 nm vesicles. Although widely used, there is evidence that ultracentrifugation may induce vesicle aggregation [82], and further, that protein contaminants may be co-isolated with EVs [83]. A theoretical analysis of ultracentrifugation by Livshits and colleagues [79] also highlighted that variability in the pelleting efficiency of different rotors can lead to variable sample recovery using this technique. Density gradient ultracentrifugation is an extension of this method, where samples are subjected to overnight centrifugation on a sucrose or iodixanol gradient [84]. This method is generally effective at separating EVs from other contaminants [85, 86], however, it is laborious and may lead to sample loss [87]. Size exclusion chromatography (SEC) methods have also been adapted for EV enrichment. Lobb and colleagues [88] assessed this method and found it to perform similarly to density gradient ultracentrifugation in terms of isolate purity. The use of commercial polymer-based reagents such as *ExoQuick* and *Invitrogen Total Exosome Isolation Kit* expedite the isolation process and avoid high speed centrifugation, however, the purity of EVs produced by these methods is generally poor [89]. Immunoaffinity based methods allow the isolation of EVs bearing specific surface markers, enabling the interrogation of EV subpopulations of interest [90]. This method generally produces pure and homogenous yields [84, 86], however, performance is highly dependent on the antibody used for capture.

Ultimately, the most appropriate EV isolation technique will depend on the sample type, the purpose of the investigation, the downstream analyses to be performed and the available equipment and resources. It is important for sample handling workflows to be individually evaluated and optimised with respect to isolate yield and quality. Increased stringency in the evaluation and reporting of EV isolation protocols will serve to increase experimental reproducibility and better facilitate inter-study comparisons.

### Physical and molecular characterisation of EVs

The utility of tumour-EV biomarker studies is underpinned by the ability to accurately determine sample characteristics, including size distribution, concentration and the molecular contents. The nature of EV samples, however, presents some unique challenges for physical and molecular characterisation. Physically, EVs exist in the sub-100 nm range and are heterogenous in size, which limits the applicability of conventional nanoparticle characterisation techniques. Further, molecular characterisation is complicated by the difficulty in isolating a highly pure EV population devoid of protein and nucleic acids from non-EV sources. The following section will discuss some of the specific factors to consider for EV characterisation.

Physical characterisation of EVs is considered an important experimental step to verify that vesicle size distribution and concentration are as expected for the sample. Platforms commonly employed for measurement include transmission electron microscopy (TEM), dynamic light scattering (DLS), nanoparticle tracking analysis (NTA) [91], flow cytometry [92] and tunable resistive pulse sensing (TRPS) [92–94]. There are several general and platform-specific issues to consider when interpreting and reporting EV measurements, which will briefly be summarised here. For a more detailed discussion of the various platforms employed for EV characterisation, refer to an investigation by Van der Pol and colleagues [95].

TEM is arguably the ‘gold standard’ technique for physical characterisation of EVs. This technique allows direct visualisation of the size and morphology of single vesicles with a resolution of ≤ 1 nm [95]. It has been suggested, however, that artefacts may be introduced during sample preparation and fixing, including vesicle shrinkage [92]. To counter this, cryo-electron microscopy (cryo-TEM) has become widely used in EV research [96, 97]. Unlike conventional TEM, cryo-TEM samples do not require staining and fixing [96, 97]. This is thought to better preserve vesicle morphology, allowing visualisation of native EV structure [98]. Both TEM and cryo-TEM are largely qualitative methods, as the number of vesicles which can be analysed is limited [99]. Flow cytometry is a technique conventionally used for single cell analysis which has been adapted for EV characterisation [100, 101]. The EV sample is focused into a narrow stream and passes through a laser beam, with the light scattering and fluorescence profile of each vesicle individually detected and recorded [101, 102]. This can be used to determine individual EV size and/or verify the presence of fluorescently labelled molecules of interest, and has been suggested as a way to interrogate the heterogeneity within EV populations [100, 103]. There are, however, currently several practical limitations of this technique. Critically, the small size and low refractive index of EVs means they generally do not scatter enough light to trigger detection on a conventional flow cytometer, with only clusters of multiple EVs and very large EVs detected [101, 102]. Several investigations have demonstrated successful triggering from fluorescence, by uniformly labelling EVs with a general membrane or protein dye [103, 104]. Care must be taken, however, to remove any unincorporated dye which can give a non-specific signal [104]. Further, achieving sufficiently bright immunofluorescent labelling of EV-associated markers can be challenging as a single EV contains a limited number of target molecules [100].

Unlike EM and flow cytometry, DLS, NTA and TRPS are all bulk measurement techniques. DLS generates size distribution information based on fluctuations in the intensity of measured light over time due to Brownian motion [105]. This technique enables rapid and bulk sample characterisation, however, there is a propensity of this technique to over-represent larger particles in the sample as these dominate the light scattering signal [105]. This must be taken into consideration when interpreting measurements of physically heterogeneous samples such as EVs. NTA builds a size distribution by tracking the Brownian motion of individual particles, and is therefore less affected by outliers than DLS [91]. It is similarly rapid, enabling the measurement of thousands of single EVs over a few minutes [106]. It is worthwhile to note, however, that it is difficult to determine the lower size limit for EVs that are reliably detected and tracked using this system. The limit of detection is dependent on both the refractive index of the particles and the suspending fluid, with previous estimates for EVs ranging between 50 and 90 nm [91, 92]. Robustly defining the limits of detection for a system is important to ensure that size and concentration information are based on true, confident EV detection and not confounded by system noise. TRPS is a non-optical measurement technique based on the electrical impedance induced by a particle as it traverses a conical nanopore [105]. This system generally requires an expert user to operate, as number of user-defined parameters must be optimised for each measurement [94]. Instrument setup and the limit of detection varies between measurements, and can be empirically determined as described in [94].

Molecular characterisation of EVs is typically achieved using conventional nucleic acid and protein analysis techniques. For evaluation of RNA, the most commonly used methods are reverse transcription PCR (RT-PCR) to detect transcripts of interest [61, 72, 107] or RNA and miRNA sequencing to obtain the full transcriptome [67, 108]. Importantly, however, the observed miRNA and mRNA profiles have been reported to be influenced by the EV extraction method and the RNA isolation protocol [77, 109], and inter-sample comparisons should be conducted with regards to this. Eldh and colleagues [109] observed that due to the differences between cellular and EV membranes that cellular RNA extraction protocols may require some modification for optimal performance. Further, Akers et al. [64] noted that transcripts conventionally used for normalisation such as the housekeeping genes GAPDH and 18S rRNA may not reliably correlate with EV RNA yield, and an alternate method of normalisation should be employed.

As for RNA, conventional analysis techniques are typically employed to characterise EV protein cargo. Detection of a small number of pre-determined protein targets is typically achieved by Western blot, using a standard sample preparation workflow as described by Choi et al. [85]. Where characterisation of the full EV proteome is required, such as for biomarker discovery, liquid chromatography tandem–mass spectrometry (LC–MS/MS) methods have been used [110, 111]. There are several challenges for characterisation of EVs by LC–MS/MS, notably the difficulty in depleting the non-vesicular protein components from complex samples which mask detection of less abundant EV associated proteins [112]. This is particularly challenging when working with protein-rich biological fluids, such as serum or plasma. For proteomic studies, therefore, the EV isolation method is critically important. A detailed discussion of the issues surrounding proteomic analysis of EVs is presented by Abramowicz et al. [112].

All of the aforementioned characterisation methods are performed on the total EV population, which is likely to comprise exosomes, microvesicles and other non-vesicular components. As previously mentioned, EV isolation and characterisation techniques do not allow the user to concretely distinguish between these and so reliably attributing physical and molecular properties to a particular EV class is difficult. Further, it can be difficult to ascertain if identified proteins and nucleic acids are true EV cargo [80]. It is important to establish that the molecules of interest are truly contained within EVs to ensure that they are reproducibly enriched during EV sample processing, as opposed to the stochastic enrichment of co-isolates such as serum proteins and circulating nucleic acids. In general, selection of the most appropriate characterisation methodologies will depend on the purpose of the investigation, as well as the equipment and expertise available.

### Translation of EV biomarkers to a clinical setting

There are specific challenges to be addressed before the potential of tumour-EV biomarkers can be realised in a clinical setting. There are several specific issues related to the collection of circulating EVs from human subjects. The level of circulating EVs is known to be influenced by numerous factors, including the time of day when the sample is collected [113] and by physical activity undertaken prior to collection [114]. These factors may influence the subsequent analysis. In addition, György and colleagues [115] have observed that after blood collection, some cells may continue to produce vesicles in vitro. They reported that the level of artefactual vesiculation was dependent on the type of tube used for blood collection. In total, Mora and colleagues [116] point out that for routine ‘biobanking’ of EVs to be feasible that collection, isolation and storage protocols would need to be thoroughly optimised and standardised. Most EV investigations to date have centred on in vitro cell line models of disease, with limited numbers of clinical samples subjected to analysis. The feasibility of high throughput isolation of tumour-EVs from complex biological fluids has therefore yet to be demonstrated. This demonstration, as well as continued evaluation and improvement of EV sample handling and characterisation methods, is warranted to continue to progress the use of tumour-EV biomarkers towards clinical applications.

## Conclusions

It is now apparent that EVs participate in a range of physiological processes and represent an important intercellular communication mechanism. There is much evidence that tumour-EVs carry tumour-associated cargo, and that they actively facilitate cancer growth. Their potential as readily accessible cancer biomarkers has been explored across a number of different contexts. There are, however, still several issues to be addressed before tumour-EV biomarkers can be considered truly feasible in a clinical setting. Currently, there is a lack of standardisation of methods for sample handling and characterisation, limiting experimental reproducibility and inter-study comparisons. In addition, there are a limited number of studies where the processing and characterisation of EVs from a large number of complex samples has been demonstrated. Nonetheless, it is evident that tumour-EVs are very promising candidate biomarkers and this field of research is likely to continue to attract much interest.

## Abbreviations

EVs: extracellular vesicles
MVs: microvesicles
MVE: multi-vesicular element
ESCRT: endosomal complex required for transport
MHC-I/MHC-II: major histocompatibility complex class 1/class 2
DCs: dendritic cells
BMDCs: bone marrow derived cells
AML: acute myeloid leukaemia
SEC: size exclusion chromatography
DLS: dynamic light scattering
NTA: nanoparticle tracking analysis
EM: electron microscopy
TRPS: tunable resistive pulse sensing
RT-PCR: reverse transcription polymerase chain reaction
LC–MS/MS: liquid chromatography tandem–mass spectrometry
